# Anti-platelet Therapy Is Associated With Lower Risk of Dementia in Patients With Cerebral Small Vessel Disease

**DOI:** 10.3389/fnagi.2022.788407

**Published:** 2022-03-31

**Authors:** Dong Pan, Xiaoming Rong, Honghong Li, Zhenhong Deng, Jia Wang, Xiaohuan Liu, Lei He, Yongteng Xu, Yamei Tang

**Affiliations:** ^1^Department of Neurology, Sun Yat-sen Memorial Hospital, Sun Yat-sen University, Guangzhou, China; ^2^Guangdong Provincial Key Laboratory of Malignant Tumor Epigenetics and Gene Regulation, Sun Yat-sen Memorial Hospital, Sun Yat-sen University, Guangzhou, China; ^3^Guangdong Province Key Laboratory of Brain Function and Disease, Zhongshan School of Medicine, Sun Yat-sen University, Guangzhou, China

**Keywords:** cerebral small vessel disease, dementia, anti-platelet therapy, clopidogrel, aspirin, cilostazol

## Abstract

**Introduction:**

Cerebral small vessel disease (CSVD) is common among older people and it could lead to dementia. Whether anti-platelet therapy (APT) could retard the cognitive decline of CSVD is unclear. The aim of the study was to evaluate, in newly diagnosed CSVD patients without dementia, the association between the APT and dementia during follow-up.

**Methods:**

We conducted a nested case-control study within a CSVD cohort. Dementia cases, such as vascular dementia (VaD), Alzheimer’s disease (AD), and unspecified dementia (UD), were individually matched (1:1) to controls by age, sex, and follow-up time. Conditional logistic regression models were used to estimate the odds ratios (*OR*s) between APT and dementia.

**Results:**

Of 9,991 patients in a cohort screened from January 2009 to December 2019 and followed-up until November 2020, 131 dementia cases were finally included and successfully matched to 131 controls. Among 262 patients with CSVD, the mean [standard deviation (SD)] age was 73.9 (7.9) years and 126 (48.1%) were men. The median [interquartile range (IQR)] follow-up periods were 4.73 (2.70–6.57) years in the control group and 2.94 (1.34–4.89) years in the case group. According to MRI at baseline, the case group showed higher CSVD burden in lacune(s) (*p* = 0.001), moderate-to-severe white matter hyperintensities (WMHs) (*p* = 0.015), enlarged perivascular spaces (EPVSs) in basal ganglia (*p* = 0.005), and brain atrophy (*p* < 0.001). The APT was associated with the lower overall dementia risk and the matched *OR* was statistically significant (a*OR* 0.15, 95% *CI* 0.05–0.45, *p* = 0.001), and clopidogrel showed protective effects on overall dementia (a*OR* 0.30, 95% *CI* 0.14–0.62, *p* = 0.001).

**Conclusion:**

Among newly diagnosed CSVD patients without dementia, APT was associated with a lower risk of dementia and clopidogrel might be an appropriate candidate in preventing dementia.

## Introduction

Cerebral small vessel disease (CSVD), a disorder of cerebral arterioles, capillaries, and venules, is one of the most common causes of dementia in older people, and usually presents with dizziness, memory decline, the weakness or numbness of limbs, mood disorders, gait dysfunction, and a general decline in function (Vermeer et al., 2003; Inzitari et al., 2009; Pantoni, 2010; Cannistraro et al., 2019). The neuroimaging CSVD features include recent small subcortical infarcts, lacunes, white matter hyperintensities (WMHs), enlarged perivascular spaces (EPVSs), cerebral microbleeds (CMBs), and brain atrophy (Wardlaw et al., 2013). The prevalence of CSVD varies with different races, age groups, and detection methods (with MRI being more sensitive than CT and 3-T MRI being more sensitive than 1.5-T MRI). The *Rotterdam Scan Study* had showed the increased median volume of white matter lesions along with increased age, and the proportion of persons without any white matter lesions is only 2.0% in persons aged 75 years or older (Vernooij et al., 2007). With continual improvement in life expectancy, a higher incidence of CSVD and associated dementia may be anticipated (Bos et al., 2018). Due to the limited understanding of etiopathogenesis in CSVD, however, there is no effective agent for CSVD and CSVD-associated dementia yet (Pedder et al., 2014; Shi and Wardlaw, 2016; Cannistraro et al., 2019).

Optimal medical management to prevent dementia are needed for aging population. A pool analysis of randomized trials by Kwok et al. (2015) showed that any of the single anti-platelet agents reduced the risk of recurrent stroke by 30% after lacunar stroke. As vascular pathology might contribute to cognitive decline, it could be inferred that anti-platelet therapy (APT) would reduce the risk of dementia in CSVD by retarding the vascular pathophysiologic process (Fujita et al., 2005; Chabriat and Bousser, 2006; Li et al., 2020). However, whether APT is effective for CSVD patients on preventing dementia remains unclear.

Since the diagnosis of CSVD relies on specific magnetic resonance (MR) sequences (Wardlaw et al., 2013), it is difficult to identify patients with CSVD by clinical symptoms, signs, and simple laboratory tests. On the other hand, the trial of CSVD demands a large number of participants and long enough observation period for cumulating clinical events (Schmidt et al., 2012), such as stroke and dementia, hence it will cost a lot on patients screening for a prospective study. The case-control study can yield important findings in shorter time and little money and efforts as compared with other study designs (Schulz and Grimes, 2002). However, the classical case-control study usually collects data cross-sectionally and thus the causal inference is always unreliable.

To address the evidence gap and avoid the paradox that might confuse causes and outcomes, we conducted a nested case-control study within a defined cohort (Ernster, 1994), which had included probable CSVD patients without dementia at baseline, to investigate whether the APT before the onset of dementia is associated with a lower risk of overall dementia during follow-up.

## Materials and Methods

### Study Design and Data Source

We conducted this nested case-control study within a historical cohort established at Sun Yat-sen Memorial Hospital, Sun Yat-sen University in Guangzhou, China, among patients with CSVD. The cohort included all consecutive “probable CSVD” patients without dementia hospitalized in our center from January 2009 to December 2019, identified by diagnostic labels, such as “lacunar infarcts,” “small subcortical brain infarcts,” “subcortical arteriosclerotic encephalopathy,” “white matter change,” “WMH,” and “Leukoaraiosis,” and followed-up until November 2020.

For this single-center historical cohort, data were extracted from the electronic hospital information system. The baseline was defined as the first-time diagnosis of CSVD. Trained medical records reviewers gathered baseline information, such as demographic characteristics (birthdate, sex, cigarette smoking, and alcohol consumption), medical history (neurological symptoms and self-reported co-existing disorders), laboratory tests on organ function (blood routine and biochemical tests), brain MR images (evaluating CSVD burden), treatment schedules (anti-platelet agents, anti-hypertension agents, and statins), and dementia outcomes. To minimize missing inputs and allow for quality control, study data were collected and managed using the Research Electronic Data Capture (REDCap) electronic data capture tools hosted at Sun Yat-sen Memorial Hospital (Harris et al., 2009, 2019).

The study was approved by the Ethics Committee of Sun Yat-sen Memorial Hospital, Sun Yat-sen University. Written informed consents were waived due to retrospective design. This study followed the Strengthening the Reporting of Observational Studies in Epidemiology (STROBE) reporting guideline, and the checklist of items is available in Supplementary Appendix 1 (von Elm et al., 2007).

### Cases and Controls

The CSVD diagnosis of each patient in both the case and control groups would be re-confirmed by both neurologists and radiologists, and those who were initially identified by diagnostic labels yet not actual CSVD would be excluded. The criteria were as follows: (1) typical imaging feature(s) of CSVD, such as lacune(s), WMH, EPVS, and brain atrophy; (2) irrespective of whether the patients exhibited neurological symptoms or not; (3) hereditary or other specific-cause of CSVD and other diseases, which showed similar CSVD change on MRI, were excluded.

Cases were defined as CSVD individuals with subsequently developing dementia during follow-up, and dementia referred specifically to vascular dementia (VaD), Alzheimer’s disease (AD), and unspecified dementia (UD), following International Classification of Diseases (ICD)-10 criteria. The diagnosis of dementia was derived from individual medical records and re-confirmed by the research physician according to the patient’s clinical manifestation, MRI, and prescription. Patients were considered as incident dementia cases if they were diagnosed with dementia or prescribed anti-dementia drugs (only Donepezil and Memantine were considered), whichever came first. Patients were included in the case group if they: (1) were aged 50 years or older at baseline; (2) were confirmed CSVD; (3) had a normal cognitive function or mild cognitive impairment at baseline; and (4) were diagnosed with dementia mentioned above during follow-up. We excluded patients who had: (1) unavailable brain MR images; (2) unavailable detailed baseline profiles; and (3) a history of stroke, irrespective of ischemic or hemorrhagic stroke. Since the study was exploratory, the sample size calculation had been omitted, and we had planned to include as many participants as possible.

Controls were manually selected from the remaining patients in the cohort who had no dementia in the follow-up. Each eligible case was matched to only one control by age at baseline (±2 years), sex, and follow-up time (the follow-up period of control was required to be longer than that of the case), and the CSVD diagnosis of each control subject was re-confirmed as well. Researchers who conducted controls selection were blinded to the drug exposures of eligible subjects.

### Drug Exposures

This study was set out to explore whether APT could reduce the risk of overall dementia among patients with CSVD. The baseline APT schedule of each patient was acquired by medical records review. On account that the treatment schedules could be adjusted at any time during the follow-up period according to disease conditions and we failed to obtain these data, receiving any APT agent at baseline would be considered as exposure, no matter how long APT lasted. In addition, we recorded the specific kinds of APT agents for further analyzing whether the effects of various drugs on dementia outcomes were different. The medical records reviewers were blinded to the group allocations.

### Measurement of Cerebral Small Vessel Disease Burden

We evaluated the imaging markers of CSVD on brain MRI at baseline obtained by using 1.5-T scanners, and sequences included axial T1-weighted, T2-weighted, and fluid-attenuated inversion recovery (FLAIR) sequences, while the recent small subcortical infarct and CMBs were not included in our study due to insufficient MRIs, such as T2-weighted gradient-recalled echo (T2-GRE), susceptibility-weighted imaging (SWI), or diffusion-weighted imaging (DWI). The detailed acquisition parameters of MRI sequences are available in Supplementary Appendix. Following the *STandards for ReportIng Vascular changes on nEuroimaging* standards (Wardlaw et al., 2013), all MRIs were assessed by an experienced neuroradiologist who was blinded to patients’ exposures and outcomes. The lacune, specifically referred to the lacunes of presumed vascular origin, was defined as a round or ovoid, subcortical, fluid-filled cavity, of 3–15 mm in diameter; on FLAIR images, lacunes generally had a central cerebrospinal fluid (CSF)-like hypo-intensity with or without a surrounding rim of hyperintensity. WMH were hyperintense on T2-weighted sequences and appeared as isointense or hypointense on T1-weighted sequences, and the changes in the deep gray matter or brainstem were not included in the category of WMH. Deep and periventricular WMH were both coded according to the Fazekas scale (total points ranging from 0 to 6) on the T2-FLAIR sequence (Fazekas et al., 1987), and moderate-to-severe WMH was defined as ≥3 points. EPVSs were fluid-filled spaces that had signal intensity similar to that of CSF on all sequences (WMHs were hyperintense while EPVSs were hypointense on FLAIR imaging) and appeared linear or small (diameters <3 mm) punctate following the course of a vessel as it went through gray or white matter. In this study, only the EPVSs in basal ganglia were counted (Potter et al., 2015). Brain atrophy was defined as a lower brain volume that was not related to trauma or infarction, which was assumed from the enlargement of sulci and ventricles (Wardlaw et al., 2013). A neuroradiologist who was unaware of drug exposures and dementia outcomes rated the above four imaging markers on brain MRIs, and we additionally scored the individual CSVD burden (range 0–5): the presence of lacunes (one point if present), the presence of brain atrophy (one point if present), the presence of moderate WMH (one point if present) or sever WMH (two points if present), and the presence of EPVS (one point if the total number of basal ganglia EPVS ≥10).

### Statistical Analysis

Continuous variables conforming to normal distribution were described by mean and standard deviation (SD) and those not conforming to normal distribution were described by the median and interquartile range (IQR). Categorical variables were described by numbers and proportions. The univariate comparisons of baseline features between groups were performed using paired *t*, Wilcoxon paired-samples signed-rank, or McNemar’s tests according to the type and distribution of variables. In addition, we described the baseline characteristics of 131 cases grouped by the specific types of dementia and performed univariate comparisons between three groups as well.

We used the *clogit* function of *survival* package in R software to construct conditional logistic regression models for adjusting the confounders on dementia outcomes and estimating odds ratios (*OR*s) and their 95% confidence intervals (95% *CI*) (Kuo et al., 2018). The crude analysis would adjust only for matched covariates, namely, age (continuous) and sex (male or female), while the multivariable-adjusted conditional logistic regression model would include in all probable confounders. Probable confounders were identified as age (continuous), sex (male or female), the history of coronary heart disease (with or without), the history of hypertension (with or without), the history of diabetes (with or without), anti-hypertension agents use (with or without), statins use (with or without), the presence of symptoms (asymptomatic or symptomatic), carotid plaques (with or without), and CSVD burdens: lacune (none or ≥1), WMHs (mild or moderate-to-severe), basal ganglia EPVSs (<10 or ≥10), and brain atrophy (with or without). The directed acyclic graph illustrated the confounders selection in Supplementary Appendix 2 (Shrier and Platt, 2008). There was no missing value in the above covariates. In the main analysis, the CSVD burden score was included in the regression model as a continuous covariate, while four imaging markers were independently included in the regression model (i.e., lacunes, WMH, EPVS, and brain atrophy) in the sensitivity analysis to assess the robustness of the result. The details of regression models were available in Supplementary Appendixes 3, 4.

All the values of *p* were reported as two-sided tests with significance defined as *p* < 0.05. Statistical analyses were performed in the R software (version 4.0.3, R Core Team^1^).

## Results

**FIGURE 1:**
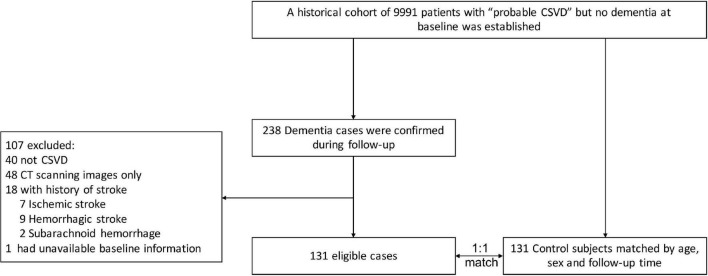
Study flowchart. The CSVD diagnosis of each patient, both in the case and control groups, was re-confirmed by neurologists and radiologists. CSVD, cerebral small vessel disease; CT, computed tomography.

Of 9,991 initially screened patients in the cohort, 238 patients were diagnosed with dementia, of whom 107 patients were excluded (40 did not have CSVD, 48 had CT scanning images only, 18 had a history of stroke, 1 patient had unavailable baseline information). A total of 131 eligible cases and 131 successfully matched controls were finally included after reviewing the medical records and MR images (Figure 1).

### Baseline Characteristics

Among the 262 patients (131 cases and 131 controls), the mean age was 73.9 (7.9 SD) years and 126 (48.1%) were men. Patients in the control group were not different (vs. case group) in age (mean ± SD years, 73.8 ± 7.8 vs. 73.9 ± 7.9, *p* = 0.548) and sex [63 (48.1%) men vs. 63 (48.1%) men, *p* > 0.999], and had longer follow-up periods [median years, 4.73 (IQR 2.70–6.57) vs. 2.94 (QR 1.34–4.89), *p* < 0.001].

The baseline characteristics of all 262 patients and groups were displayed in Tables 1, 2. Patients in the case group (vs. the control group) were less often asymptomatic [9 (6.9%) vs. 22 (16.8%), *p* = 0.031] and more often presenting with carotid plaques [89 (67.9%) vs. 66 (50.4%), *p* = 0.003], while co-existing disorders (atrial fibrillation, coronary heart disease, hypertension, and diabetes) showed no significant differences between groups. According to MRI at baseline, the case group showed higher CSVD burden in lacune(s) [60 (45.8%) vs. 34 (26.0%), *p* = 0.001], moderate-to-severe WMH [80 (61.1%) vs. 61 (46.6%), *p* = 0.015], basal ganglia EPVS [35 (26.7%) vs. 18 (13.7%), *p* = 0.005], and brain atrophy [96 (73.3%) vs. 54 (41.2%), *p* < 0.001] as compared with the control group (Table 1). The distribution of CSVD burden score was displayed in Supplementary Appendix 5. The baseline features of 131 cases categorized by the subtypes of dementia were basically balanced except that VaD cases appeared to have a higher proportion of receiving anti-hypertension therapy [VaD vs. AD vs. UD group, 34 of 43 (79.1%) vs. 30 of 55 (54.5%) vs. 19 of 33 (57.6%), *p* = 0.032; Table 2].

**TABLE 1:** The baseline characteristics of study population.

	All patients	Control group	Case group	*p*-Values
	*N* = 262	*N* = 131	*N* = 131	
Sex – male, no. (%)	126 (48.1)	63 (48.1)	63 (48.1)	>0.999
Age – years, mean (SD)	73.9 (7.9)	73.8 (7.8)	73.9 (7.9)	0.548
Follow-up period – years, median (IQR)	3.86 (1.97, 5.77)	4.73 (2.70, 6.57)	2.94 (1.34, 4.89)	<0.001
**Symptoms – with, no. (%)**				
Asymptomatic	31 (11.8)	22 (16.8)	9 (6.9)	0.031
Dizziness	140 (53.4)	79 (60.3)	61 (46.6)	0.035
Weakness of limbs	73 (27.9)	33 (25.2)	40 (30.5)	0.362
Numbness of limbs	27 (10.3)	11 (8.40)	16 (12.2)	0.424
Bladder-bowel dysfunction	43 (16.4)	15 (11.5)	28 (21.4)	0.037
Headache	14 (5.3)	5 (3.8)	9 (6.9)	0.423
Memory decline	12 (4.6)	2 (1.5)	10 (7.6)	0.043
Gait disorder	10 (3.8)	5 (3.8)	5 (3.8)	>0.999
Vertigo	10 (3.8)	8 (6.1)	2 (1.5)	0.114
Sleep disorder	3 (1.2)	0 (0.0)	3 (2.3)	0.248
**Co-existing disorders – with, no. (%)**				
Atrial fibrillation	6 (2.3)	2 (1.5)	4 (3.1)	0.683
Coronary heart disease	47 (17.9)	19 (14.5)	28 (21.4)	0.176
Hypertension	171 (65.3)	87 (66.4)	84 (64.1)	0.680
Diabetes	82 (31.3)	36 (27.5)	46 (35.1)	0.174
Cigarette smoking – with, no. (%)	37 (14.1)	21 (16.0)	16 (12.2)	0.441
Alcohol consumption – with, no. (%)	15 (5.7)	11 (8.4)	4 (3.1)	0.121
Carotid plaque – with, no. (%)	155 (59.2)	66 (50.4)	89 (67.9)	0.003
**Medications – with, no. (%)**				
Anti-platelet drugs	215 (82.1)	112 (85.5)	103 (78.6)	0.164
Anti-hypertension drugs	168 (64.1)	85 (64.9)	83 (63.4)	0.806
Statins	155 (59.2)	72 (55.0)	83 (63.4)	0.124
**Brain MRI findings – with, no. (%)**				
Lacune ≥1	94 (35.9)	34 (26.0)	60 (45.8)	0.001
Moderate-to-severe WMH	141 (53.8)	61 (46.6)	80 (61.1)	0.015
Basal ganglia EPVS ≥10	53 (20.2)	18 (13.7)	35 (26.7)	0.005
Brain atrophy	150 (57.3)	54 (41.2)	96 (73.3)	<0.001

*WMH, white matter hyperintensities; EPVS, enlarged perivascular spaces.*

**TABLE 2:** The baseline characteristics of 131 cases grouped by specific dementia.

	VaD group	AD group	UD group	*p-*Values
	*N* = 43	*N* = 55	*N* = 33	
Sex – male, no. (%)	25 (58.1)	25 (45.5)	13 (39.4)	0.235
Age – years, mean (SD)	73.2 (7.7)	74.5 (8.3)	73.8 (7.7)	0.728
Follow-up period – years, median (IQR)	2.89 (1.21, 4.76)	3.61 (1.68, 4.89)	2.55 (1.05, 5.05)	0.464
**Symptoms – with, no. (%)**				
Asymptomatic	2 (4.7)	7 (12.7)	0 (0.0)	0.072
Dizziness	19 (44.2)	26 (47.3)	16 (48.5)	0.924
Weakness of limbs	9 (20.9)	4 (7.3)	3 (9.1)	0.128
Numbness of limbs	14 (32.6)	13 (23.6)	13 (39.4)	0.281
Bladder-bowel dysfunction	13 (30.2)	9 (16.4)	6 (18.2)	0.220
Headache	1 (2.3)	7 (12.7)	1 (3.0)	0.107
Memory decline	2 (4.7)	5 (9.1)	3 (9.1)	0.703
Gait disorder	1 (2.3)	2 (3.6)	2 (6.1)	0.726
Vertigo	1 (2.3)	0 (0.0)	1 (3.0)	0.335
Sleep disorder	2 (4.7)	1 (1.8)	0 (0.0)	0.612
**Co-existing disorders– with, no. (%)**				
Atrial fibrillation	1 (2.3)	1 (1.8)	2 (6.1)	0.566
Coronary heart disease	13 (30.2)	10 (18.2)	5 (15.2)	0.212
Hypertension	33 (76.7)	32 (58.2)	19 (57.6)	0.109
Diabetes	22 (51.2)	14 (25.5)	10 (30.3)	0.024
Cigarette smoking – with, no. (%)	3 (7.0)	10 (18.2)	3 (9.1)	0.236
Alcohol consumption – with, no. (%)	0 (0.0)	4 (7.3)	0 (0.0)	0.089
Carotid plaque – with, no. (%)	31 (72.1)	34 (61.8)	24 (72.7)	0.442
**Medications – with, no. (%)**				
Anti-platelet drugs	39 (90.7)	39 (70.9)	25 (75.8)	0.054
Anti-hypertension drugs	34 (79.1)	30 (54.5)	19 (57.6)	0.032*
Statins	31 (72.1)	30 (54.5)	22 (66.7)	0.182
**Brain MRI findings – with, no. (%)**				
Lacune ≥1	18 (41.9)	28 (50.9)	14 (42.4)	0.607
Moderate-to-severe WMH	27 (62.8)	33 (60.0)	20 (60.6)	0.959
Basal ganglia EPVS ≥10	10 (23.3)	17 (30.9)	8 (24.2)	0.651
Brain atrophy	35 (81.4)	37 (67.3)	24 (72.7)	0.292

VaD, vascular dementia; AD, Alzheimer’s disease; UD, unspecified dementia; WMH, white matter hyperintensities; EPVS, enlarged perivascular spaces. *The difference between VaD group and AD group was significant (p = 0.018), that between VaD group and UD group was significant as well (p = 0.049); the difference between AD group and UD group was insignificant (p = 0.827). The p-values above were calculated by Fisher’s exact tests.

### Risk of Overall and Subtypes of Dementia

**FIGURE 2:**
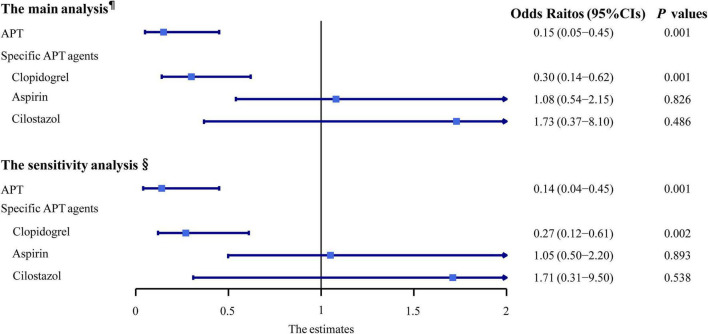
Multivariable-adjusted conditional logistic regression analyses of the association between the anti-platelet therapy and dementia. ^¶^Adjusted odds ratios (*OR*s) and their 95% *CI*s were from the conditional logistic regression model with the adjustment of CSVD burden scores (0–5 score based on MRI assessment of lacunes, WMH, EPVS, and brain atrophy) and other confounders. ^§^Adjusted *OR*s and their 95% *CI*s were from the conditional logistic regression model with the adjustment of four CSVD markers (lacune ≥1, moderate-to-severe WMH, EPVS ≥10, and brain atrophy) and other confounders. APT, anti-platelet therapy; CI, confidence interval.

There were 103 (78.6%) and 112 (85.5%) patients receiving APT in the case and the control groups, respectively. The crude analyses of the association between the APT and dementia, adjusting only for matched covariates (sex and age), were reported in Table 3. We observed a decreased risk of overall dementia in patients receiving any APT agents, compared with those who did not, after adjusting for confounders (*OR* 0.16, 95% *CI* 0.05–0.46, *p* = 0.001; Figure 2). Furthermore, we studied the association between three different APT agents and overall dementia, and both univariate and multivariate analyses showed that clopidogrel was associated with a decreased risk of overall dementia (*OR* 0.30, 95% *CI* 0.14–0.62, *p* = 0.001; Figure 2), while aspirin (*OR* 1.09, 95% *CI* 0.55–2.17, *p* = 0.800; Figure 2) and cilostazol (*OR* 1.64, 95% *CI* 0.36–7.53, *p* = 0.526; Figure 2) showed insignificant effects. In the sensitivity analysis, APT showed similar effects on overall dementia (*OR* 0.14, 95% *CI* 0.04–0.46, *p* = 0.001; Figure 2), and clopidogrel had a protective effect as well (*OR* 0.27, 95% *CI* 0.12–0.61, *p* = 0.002; Figure 2).

**TABLE 3:** The crude analyses of the association between the anti-platelet therapy and dementia, adjusting only for matched covariates (sex and age).

	No. exposures/no. patients (%)		Odds ratio (95% CI)*	*p*-Values
	Control group	Case group		
**All dementia**				
APT	112/131 (85.5)	103/131 (78.6)	0.58 (0.28–1.18)	0.133
**Specific anti-platelet agents^†^**				
Clopidogrel	78/131 (59.5)	63/131 (48.1)	0.56 (0.32–0.98)	0.041
Aspirin	39/131 (29.8)	38/131 (29.0)	0.98 (0.54–1.78)	0.957
Cilostazol	4/131 (3.1)	8/131 (6.1)	1.95 (0.59–6.52)	0.276
**Vascular dementia**				
APT	35/43 (81.4)	39/43 (90.7)	2.50 (0.63–9.92)	0.192
**Specific anti-platelet agents^†^**				
Clopidogrel	23/43 (53.5)	24/43 (55.8)	1.13 (0.44–2.95)	0.796
Aspirin	13/43 (30.2)	13/43 (30.2)	1.07 (0.38–3.01)	0.893
Cilostazol	4/43 (9.3)	3/43 (7.0)	0.68 (0.14–3.19)	0.621
**Alzheimer’s disease**				
APT	50/55 (90.9)	39/55 (70.9)	*NA* ^¶^	0.999
**Specific anti-platelet agents^†^**				
Clopidogrel	34/55 (61.8)	22/55 (40.0)	0.28 (0.10–0.78)	0.015
Aspirin	19/55 (34.5)	18/55 (32.7)	0.90 (0.34–2.34)	0.822
Cilostazol	0/55 (0.0)	3/55 (5.5)	*NA* ^¶^	0.998
**Unspecified dementia**				
APT	27/33 (81.8)	25/33 (75.8)	0.69 (0.21–2.20)	0.528
**Specific anti-platelet agents^†^**				
Clopidogrel	21/33 (63.6)	17/33 (51.5)	0.56 (0.20–1.59)	0.276
Aspirin	7/33 (21.2)	7/33 (21.2)	0.99 (0.32–3.11)	0.988
Cilostazol	0/33 (0.0)	2/33 (6.1)	*NA* ^¶^	0.999

*Odds ratios and their 95% CIs were estimated by a conditional logistic regression model adjusting only for matched covariates (sex and age).

*^†^Individuals receiving more than one anti-platelet agent could be involved in more than one subcategory hence the total numbers could be beyond the numbers of receiving any anti-platelet agents. ^¶^The 95% CI was 0 to +∞ because the frequency of at least one observation unit in the fourfold table was zero. APT, anti-platelet therapy.*

As for three different subtypes of dementia, APT showed no significant effects in preventing dementia. However, clopidogrel showed protective effect in preventing AD (*OR* 0.19, 95% *CI* 0.05–0.77, *p* = 0.020) and UD (*OR* 0.14, 95% *CI* 0.02–0.85, *p* = 0.032) (Supplementary Appendix 6).

## Discussion

In this nested case-control study among patients with CSVD, we found that the APT was associated with a lower risk of overall dementia. Since the vascular pathology and associated risk factors could contribute to dementia, and possible mechanisms underlying CSVD involve microinfarction or embolism, vascular inflammation, endothelial dysfunction, blood–brain barrier (BBB) leakage, and subsequent cellular damage (Fujita et al., 2005; Cuadrado-Godia et al., 2018; Thrippleton et al., 2019; Li et al., 2020), we had hypothesized that the APT for newly diagnosed CSVD might prevent or retard the deleterious pathophysiological processes of cerebral small vessels, leading to improved long-term dementia outcomes. The findings of the present study support our hypothesis and suggest an association between APT and the decreased risk of overall dementia.

However, some population-based studies had come to different conclusions. In an observational cohort study in 2007 based on a randomized controlled study population, *Women’s health study*, which had included 6,377 healthy women aged 65 years or more, the long-term use of aspirin showed no overall benefits for cognition (Kang et al., 2007). In 2020, the secondary analysis of the *ASPREE study*, a double-blind, placebo-controlled study of low-dose aspirin on 19,114 participants, demonstrated that aspirin did not reduce the incidence of dementia for healthy elderly adults (Ryan et al., 2020). Another randomized, open-label trial including community-resident patients who had AD, showed no significant cognition improvement in the low-dose aspirin arm measured by the Mini Mental State Examination (MMSE) and increased the risk of serious bleeds (Bentham et al., 2008). Although these studies had provided class I to II evidence that the use of aspirin has no significant effects in improving cognitive function, there are several issues remaining unsolved. First of all, previous studies selected participants by age, not by imaging markers, while aging people are not equal to the patients with CSVD. The most of the individuals in these studies were less likely to be the typical patients with CSVD. Second, not all patients with CSVD need medical intervention because some mild white matter lesions could occasionally resolve spontaneously or be stable although the majority of them would gradually progress (Schmidt et al., 2012), hence the adverse drug effects might counteract the benefits of APT on outcomes. Furthermore, aspirin was the most frequently studied anti-platelet agent while the studies of other agents were almost empty, such as clopidogrel and cilostazol (Bath and Wardlaw, 2015; Yanai and Endo, 2019). Finally, we found that in the case group, the median time from baseline to incident dementia was approximately 3 years, which may be one of the reasons for the limited reports on the efficacy of APT in short-term follow-up studies (Jaturapatporn et al., 2012). Further, our result provides a reference for the time interval setting for longitudinal studies. In our study, we were able to select patients with CSVD from a defined cohort by medical records review and brain MRIs assessment, and the final diagnosis of each eligible subject (both the case and the control groups) was re-confirmed by neurologists and radiologists. Overall, the individuals of our study are actual CSVD patients without dementia at baseline, and we are the first to investigate the protective effects of APT for patients with CSVD on dementia in the Chinese population, to our knowledge.

In our study, to reduce the total numbers of covariates entering the regression model thus maintaining enough power to detect a significant association between APT and incident dementia, we integrated the four imaging markers (lacunes, WMH, EPVS, and brain atrophy) into a continuous variable (CSVD burden score) and included it in the regression model. Yilmaz et al. (2018) had developed a practical CSVD sum score composed by counting the presence of four MRI markers (WMH, lacunes, CMBs, and EPVS; range 0–4) in 2018. They found that a higher CSVD sum score on MRI showed stronger associations with the risk of dementia than individual markers. Whereas, we failed to analyze the impact of CMBs on dementia due to insufficient MRIs of SWI or T2-GRE sequences in this study, thus we did not utilize the method proposed by Yilmaz et al. (2018) to score the CSVD burden of our studied patients. In the main analysis, the CSVD burden score entered the model as a continuous covariate, while four above-mentioned imaging markers independently entered the model in the sensitivity analysis to assess the robustness of our findings. Although the main and the sensitivity analyses yielded similar results, we do not recommend using our method to score CSVD burden in clinical practice until further prospective studies validate the discrimination ability and clinical utility.

It is worth noting that the overall analysis of APT showed a significant protective effect of incident dementia, but it was presumably dominated by the clopidogrel use. Clopidogrel is a P2Y12 receptor blocker (P2Y12 activation is needed for platelet activation) but also acts on microglial P2Y12Rs if it crosses the BBB (Dorsam et al., 2003; Lou et al., 2016); cilostazol is a phosphodiesterase-3 (PDE3) inhibitor (Schrör, 2002), and aspirin blocks cyclooxygenase-1 (COX1) and thromboxane A2 production (Smith et al., 1980; Schrör, 1997). All of these drugs are able to inhibit platelet aggregation according to their pharmacological targets, whereas, our finding showed that, out of these 3 drugs, it was only clopidogrel that significantly reduced the risk of incident dementia, while cilostazol and aspirin appeared to have no such effect. The difference in the dementia outcome between the effects of clopidogrel vs. the effects of cilostazol and of aspirin, suggests that the inhibiting platelet aggregation might not be the only cause of the prevention of dementia. One possible explanation could be that the effect seen was a result of clopidogrel crossing the BBB and preventing microglia sending processes to the sites of endothelial cell damage or neuronal ATP release (Lou et al., 2016). In the future, both animals and population studies would be needed to explore the effects of clopidogrel on the central nervous system and to validate the efficacy and safety of clopidogrel for preventing dementia among patients with CSVD.

Another interesting finding was that the proportion of clopidogrel use was apparently higher than the other two agents in both the case and the control groups in our study, although aspirin was the most widely used anti-platelet agent in stroke worldwide. It suggests that the real-world clinical management for these patients with CSVD is quite different from the standard secondary prevention strategy of stroke (Hankey, 2014). For these patients with CSVD, who are usually present with chronic disease processes and milder clinical symptoms as compared with stroke, clinicians might have a propensity to prescribe non-aspirin agents to avoid gastrointestinal adverse effects and bleedings.

### Limitations of the Study

This study still has several limitations. First of all, although we initially screened 9,991 “probable CSVD” patients, less than 3% of them were included in the analysis due to case-control design and a quite low incidence of dementia. Potential selection bias might limit the generalizability of our findings. Second, this study only reveals an association between APT and a lower risk of dementia, and individual adherence to APT warrants further investigation, thus we cannot easily recommend that APT can be used in patients with CSVD for preventing dementia. Third, we failed to obtain enough brain MRIs at the endpoint for CSVD burden assessments, thereby the study was unable to examine the impact of the CSVD progression on the effect of APT for incident dementia. Finally, we were unable to study the effects of APT on stroke recurrence, bleedings, and other safety outcomes accounting for insufficient data, and we could not determine which subtype of dementia was more effective with APT due to the limited sample size of each subgroup.

Although we did perform multivariate regression models for adjusting confounders, we were unable to correct all potential biases. These limitations highlight the importance of randomized controlled trials focused on this patient population. Future randomized studies conducted in patients with CSVD for preventing dementia are warranted to select patients with actual high-risk CSVD based on imaging markers burden, and clopidogrel might be an appropriate candidate in such trials according to our findings.

## Conclusion

Among newly diagnosed CSVD patients without dementia, APT was associated with the lower risk of overall dementia and clopidogrel might be an appropriate candidate in preventing dementia. The study findings are limited by potential confounders due to retrospective observational design and further prospective randomized clinical trials are needed to validate these findings.

## Data Availability Statement

The datasets used and/or analyzed during the current study are available from the corresponding author (YT) upon reasonable request.

## Ethics Statement

The studies involving human participants were reviewed and approved by the Ethics Committee of Sun Yat-sen Memorial Hospital. Written informed consent for participation was not required for this study in accordance with the national legislation and the institutional requirements.

## Author Contributions

DP and YT: conception and design. DP, XR, HL, ZD, JW, XL, LH, and YX: acquisition of data. DP, XR, and YT: analysis and interpretation of data. DP, XR, HL, ZD, JW, XL, LH, YX, and YT: writing, review, and/or revision of the manuscript. YT: study supervision. All authors contributed to the article and approved the submitted version.

## Conflict of Interest

The authors declare that the research was conducted in the absence of any commercial or financial relationships that could be construed as a potential conflict of interest.

## Publisher’s Note

All claims expressed in this article are solely those of the authors and do not necessarily represent those of their affiliated organizations, or those of the publisher, the editors and the reviewers. Any product that may be evaluated in this article, or claim that may be made by its manufacturer, is not guaranteed or endorsed by the publisher.

## Footnotes

1
http://www.R-project.org/
