# Supplementary material for: Anti-platelet Therapy Is Associated With Lower Risk of Dementia in Patients With Cerebral Small Vessel Disease
Source: Front Aging Neurosci. 2022 Mar 31;14:788407. doi: 10.3389/fnagi.2022.788407 (PMC9008232; doi:10.3389/fnagi.2022.788407)
Supplement: Supplementary file 1 [file Presentation_1.pdf]

Anti-platelet therapy is associated with lower risk of dementia of age-related cerebral small vessel disease

Dong Pan†, M.D., Xiaoming Rong†, M.D. Ph.D., Honghong Li, M.D., Zhenhong Deng, M.D., Jia Wang, M.D., Xiaohuan Liu, M.D., Lei He, M.D. Ph.D., Yongteng Xu, M.D., Yamei Tang*, M.D. Ph.D.

Supplementary Appendix

Supplementary Appendix S1: STROBE Statement—Checklist of items that should be included in reports of case-control studies......2

Supplementary Appendix S2: The directed acyclic graph illustrating confounders selection......4

Supplementary Appendix S3: The conditional logistic regression model for the estimates of anti-platelet therapy for overall dementia. 5

Table S3.1 The model in the main analysis......5

Table S3.2 The model in the sensitivity analysis. 6

Supplementary Appendix S4: The conditional logistic regression model for the estimates of clopidogrel use for overall dementia......7

Table S4.1 The model in the main analysis......7

Table S4.2 The model in the sensitivity analysis. 8

Supplementary Appendix S5: CSVD burden distribution between groups. 9

Supplementary Appendix S6: The multivariable conditional logistic regression analyses on the association between APT and subtypes of dementia......10

Supplementary Appendix S7: The detailed acquisition information of MRI sequences. 11

Supplementary Appendix S1: STROBE Statement—Checklist of items that should be included in reports of case-control studies.

	Item No	Recommendation	Locations
**Title and abstract**	1	(*a*) Indicate the study's design with a commonly used term in the title or the abstract	Page 4
		(*b*) Provide in the abstract an informative and balanced summary of what was done and what was found	Page 4
**Introduction**			
Background/rationale	2	Explain the scientific background and rationale for the investigation being reported	Page 6
Objectives	3	State specific objectives, including any prespecified hypotheses	Page 7
**Methods**			
Study design	4	Present key elements of study design early in the paper	Page 7
Setting	5	Describe the setting, locations, and relevant dates, including periods of recruitment, exposure, follow-up, and data collection	Page 7
Participants	6	(*a*) Give the eligibility criteria, and the sources and methods of case ascertainment and control selection. Give the rationale for the choice of cases and controls	Page 8
		(*b*) For matched studies, give matching criteria and the number of controls per case	Page 8-9
Variables	7	Clearly define all outcomes, exposures, predictors, potential confounders, and effect modifiers. Give diagnostic criteria, if applicable	Page 9
Data sources/ measurement	8*	For each variable of interest, give sources of data and details of methods of assessment (measurement). Describe comparability of assessment methods if there is more than one group	Page 7<br>Page 9
Bias	9	Describe any efforts to address potential sources of bias	Line 149<br>Line 156<br>Line 175
Study size	10	Explain how the study size was arrived at	Line 144
Quantitative variables	11	Explain how quantitative variables were handled in the analyses. If applicable, describe which groupings were chosen and why	NA
Statistical methods	12	(*a*) Describe all statistical methods, including those used to control for confounding	Page 10-11
		(*b*) Describe any methods used to examine subgroups and interactions	
		(*c*) Explain how missing data were addressed	
		(*d*) If applicable, explain how matching of cases and controls was addressed	
		(*e*) Describe any sensitivity analyses	
**Results**			
Participants	13*	(a) Report numbers of individuals at each stage of study—eg numbers potentially eligible, examined for eligibility, confirmed eligible, included in the study, completing follow-up, and analysed	Line 204-207, Page 11
		(b) Give reasons for non-participation at each stage	
		(c) Consider use of a flow diagram	Figure 1
Descriptive data	14*	(a) Give characteristics of study participants (eg demographic, clinical, social) and information on exposures and potential confounders	Table 1-2
		(b) Indicate number of participants with missing data for each variable of interest	Line 197
Outcome data	15*	Report numbers in each exposure category, or summary measures of exposure	Line 224

Main results	16	(*a*) Give unadjusted estimates and, if applicable, confounder-adjusted estimates and their precision (eg, 95% confidence interval). Make clear which confounders were adjusted for and why they were included	Table 3<br>Figure 2<br>Line 191-195
		(*b*) Report category boundaries when continuous variables were categorized	NA
		(*c*) If relevant, consider translating estimates of relative risk into absolute risk for a meaningful time period	NA
Other analyses	17	Report other analyses done—eg analyses of subgroups and interactions, and sensitivity analyses	Line 197-200
**Discussion**			
Key results	18	Summarise key results with reference to study objectives	Page 13-16
Limitations	19	Discuss limitations of the study, taking into account sources of potential bias or imprecision. Discuss both direction and magnitude of any potential bias	
Interpretation	20	Give a cautious overall interpretation of results considering objectives, limitations, multiplicity of analyses, results from similar studies, and other relevant evidence	
Generalisability	21	Discuss the generalisability (external validity) of the study results	
**Other information**			
Funding	22	Give the source of funding and the role of the funders for the present study and, if applicable, for the original study on which the present article is based	Line 25-29

*Give information separately for cases and controls.

Note: An Explanation and Elaboration article discusses each checklist item and gives methodological background and published examples of transparent reporting. Information on the STROBE Initiative is available at http://www.strobe-statement.org.

Supplementary Appendix S2: The directed acyclic graph illustrating confounders selection.

A Directed Acyclic Graph (DAG) was used to identify potential confounders and independent risk factors. White ovals represent potential confounders and independent risk factors that were included in a multivariable conditional logistic regression model for estimating the effect of APT on outcome. CSVD burden contains white matter hyperintensities, enlarged perivascular spaces, lacunes and brain atrophy.

Abbreviations: APT, anti-platelet therapy; CSVD, cerebral small vessel disease; CHD, coronary heart disease; AHT, anti-hypertension therapy.

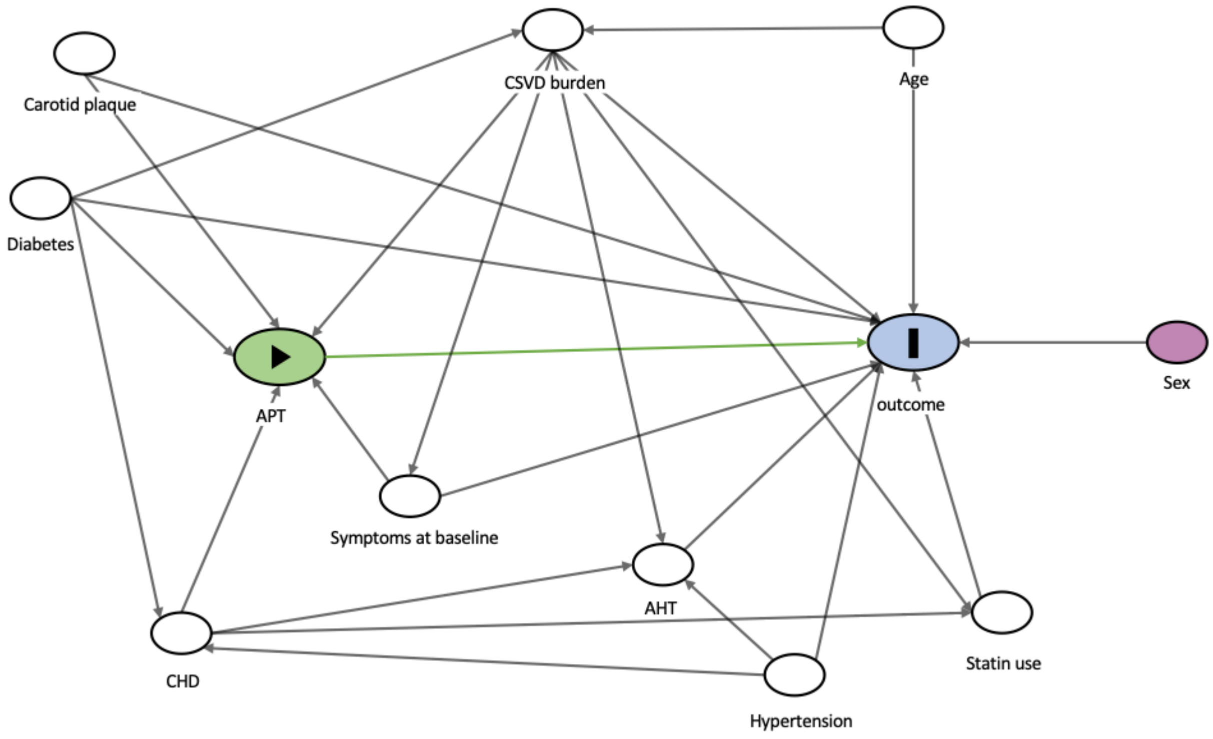

Supplementary Appendix S3: The conditional logistic regression model for the estimates of anti-platelet therapy for overall dementia.

Table S3.1 The model in the main analysis.

Predictors	Estimates	CI	P
**APT**	0.15	0.05 – 0.45	**0.001**
**Age (years)**	0.96	0.79 – 1.18	0.708
**Asymptomatic at diagnosis**	0.39	0.14 – 1.06	0.065
**History of CHD**	2.17	0.90 – 5.21	0.084
**Carotid plaque**	2.54	1.20 – 5.41	**0.015**
**AHT**	0.41	0.14 – 1.20	0.102
**Hypertension**	1.25	0.41 – 3.78	0.695
**diabetes**	1.32	0.64 – 2.75	0.451
**Statin use**	3.28	1.38 – 7.82	**0.007**
**cSVD burden score**	1.83	1.38 – 2.42	**<0.001**

Abbreviations: CI, confidence interval; APT, anti-platelet therapy; CHD, coronary heart disease; AHT, anti-hypertension therapy; cSVD, cerebral small vessel disease.

Table S3.2 The model in the sensitivity analysis.

Predictors	Estimates	CI	p
**APT**	0.14	0.04 – 0.45	**0.001**
**Age (years)**	0.95	0.77 – 1.18	0.659
**Asymptomatic at diagnosis**	0.34	0.11 – 1.02	0.054
**History of CHD**	1.76	0.67 – 4.61	0.248
**Carotid plaque**	2.76	1.19 – 6.39	**0.018**
**AHT**	0.36	0.11 – 1.17	0.089
**Hypertension**	1.58	0.47 – 5.24	0.457
**diabetes**	1.02	0.45 – 2.30	0.968
**Statin use**	3.78	1.42 – 10.02	**0.008**
**Lacune ≥1**	1.71	0.77 – 3.79	0.188
**Moderate-to-severe WMH**	0.99	0.43 – 2.29	0.989
**EPVs ≥10**	2.16	0.73 – 6.42	0.164
**Brain atrophy**	9.97	3.25 – 30.55	**<0.001**

Abbreviations: CI, confidence interval; APT, anti-platelet therapy; CHD, coronary heart disease; AHT, anti-hypertension therapy; WMH, whiter matter hyperintensities; EPVs, enlarged perivascular spaces.

Supplementary Appendix S4: The conditional logistic regression model for the estimates of clopidogrel use for overall dementia.

Table S4.1 The model in the main analysis.

Predictors	Estimates	CI	P
**Clopidogrel use**	0.30	0.14 – 0.62	**0.001**
**Age (years)**	1.02	0.84 – 1.24	0.823
**Asymptomatic at diagnosis**	0.50	0.20 – 1.25	0.140
**History of CHD**	2.19	0.91 – 5.30	0.081
**Carotid plaque**	2.37	1.14 – 4.93	**0.021**
**AHT**	0.36	0.12 – 1.08	0.069
**Hypertension**	1.44	0.47 – 4.39	0.519
**diabetes**	1.24	0.61 – 2.54	0.551
**Statin use**	2.47	1.13 – 5.40	**0.023**
**cSVD burden score**	1.79	1.35 – 2.38	**<0.001**

Abbreviations: CI, confidence interval; APT, anti-platelet therapy; CHD, coronary heart disease; AHT, anti-hypertension therapy; cSVD, cerebral small vessel disease.

Table S4.2 The model in the sensitivity analysis.

Predictors	Estimates	CI	p
**Clopidogrel use**	0.27	0.12 – 0.61	**0.002**
**Age (years)**	1.03	0.83 – 1.29	0.760
**Asymptomatic at diagnosis**	0.41	0.14 – 1.16	0.091
**History of CHD**	1.93	0.73 – 5.06	0.183
**Carotid plaque**	2.49	1.10 – 5.66	**0.029**
**AHT**	0.28	0.08 – 0.93	**0.038**
**Hypertension**	1.92	0.56 – 6.57	0.297
**diabetes**	0.92	0.41 – 2.08	0.838
**Statin use**	3.16	1.26 – 7.92	**0.014**
**Lacune ≥1**	1.72	0.79 – 3.74	0.172
**Moderate-to-severe WMH**	0.95	0.42 – 2.18	0.905
**EPVs ≥10**	2.64	0.89 – 7.86	0.082
**Brain atrophy**	9.93	3.25 – 30.34	**<0.001**

Abbreviations: CI, confidence interval; APT, anti-platelet therapy; CHD, coronary heart disease; AHT, anti-hypertension therapy; WMH, whiter matter hyperintensities; EPVs, enlarged perivascular spaces.

Supplementary Appendix S5: CSVD burden distribution between groups.

All eligible subjects were scored on cSVD burden based on four radiographic biomarkers: presence of lacunes (one point if present), presence of brain atrophy (one point if presence), presence of medium WMH (one point if present) or sever WMH (two points if presence) and presence of EPVs (one point if BGEPVs ≥10) (range 0-5).

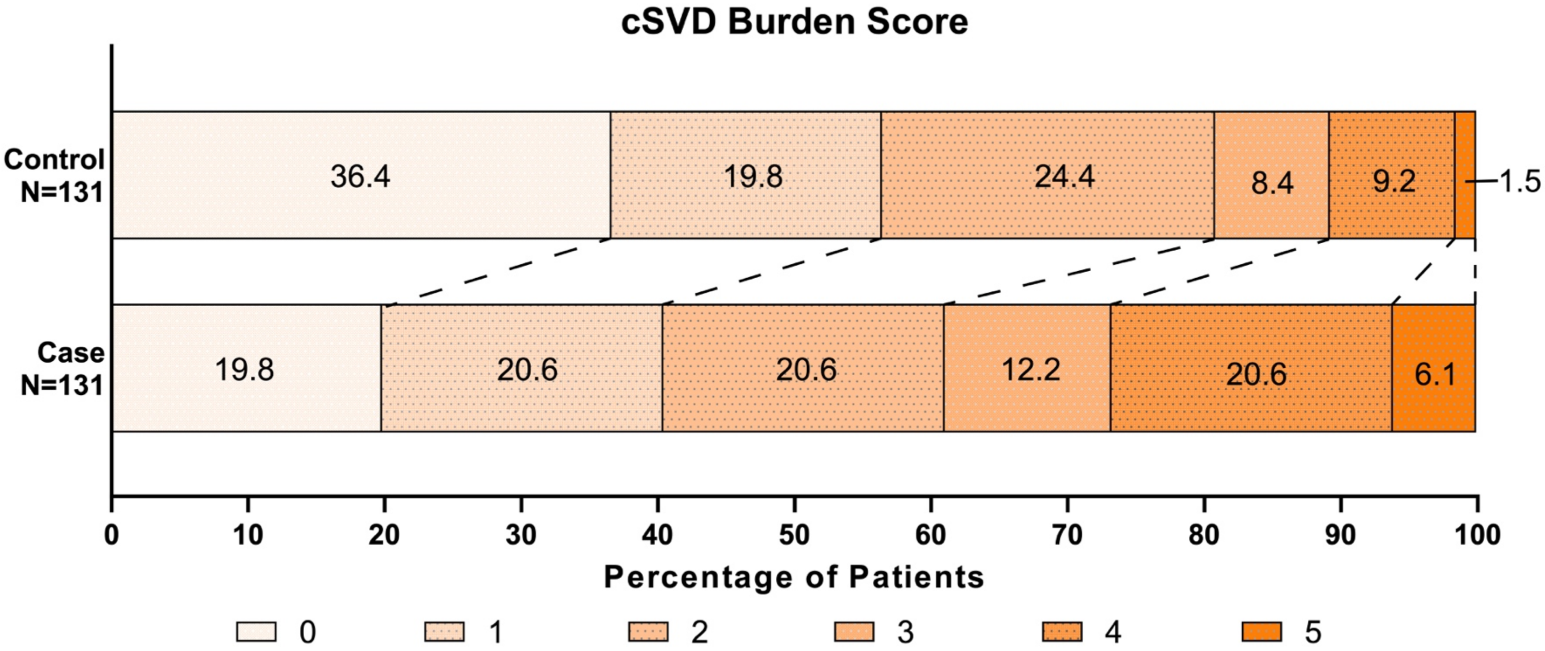

Supplementary Appendix S6: The multivariable conditional logistic regression analyses on the association between APT and subtypes of dementia.

The models adjusted for the same covariates as in the main analysis.

* The actual CI was (0.004-2.990).

£ The CIs were (0, +∞).

	OR (95% CI)	P values
**Vascular dementia**		
APT	0.11 (0.00-2.99) *	0.193
Clopidogrel	0.64 (0.10-4.14)	0.637
Aspirin	0.27 (0.03-2.15)	0.215
Cilostazol	2.68 (0.23-31.72)	0.434
**Alzheimer's diseases**		
APT	NA£	0.998
Clopidogrel	0.19 (0.05-0.77)	0.020
Aspirin	0.86 (0.25-3.04)	0.820
Cilostazol	NA£	0.999
**Unspecified dementia**		
APT	0.27 (0.03-2.26)	0.227
Clopidogrel	0.14 (0.02-0.85)	0.032
Aspirin	4.61 (0.62-34.46)	0.137
Cilostazol	NA£	0.999

Supplementary Appendix S7: The detailed acquisition parameters of MRI sequences.

MRI scanning was performed on 1.5 Tesla Siemens/Philips Magnetom Trio Tim scanner, using a 32-channel head coil, at the Sun Yat-sen Memorial Hospital, Sun Yat-sen University. FLAIR imaging was T2-weighted.

**Sequence**	**T1WI**	**T2WI**
TR (ms)	1090	4500
TE (ms)	8.4	118
Flip angle	150	150
FOV (mm)	230×208	230×208
Voxel size (mm)	0.4×0.4×5	0.2×0.2×5
Slice thickness (mm)	5	5
Overlapping gap (mm)	1	1
Acquisition time (min)	2′22″	2′58″

MRI, magnetic resonance imaging; T1-weighted imaging (T1WI), T2-weighted imaging (T2WI); TR, repetition time; TE, echo time; FOV, field of view; VIBE, volume interpolated body examination; FS, fat-suppression.
